# Mitigation of Self-p-Doping and Off-Centering Effect in Tin Perovskite via Strontium Doping

**DOI:** 10.1021/acsenergylett.4c02974

**Published:** 2024-12-31

**Authors:** Chiara Frasca, Paola Alippi, Renè Schwiddessen, Karunanantharajah Prashanthan, Giuseppe Nasti, Shengnan Zuo, Muhammad Okash Ur Rehman, Mahmoud Hussein Aldamasy, Noor Titan Putri Hartono, Artem Musiienko, Antonio Abate

**Affiliations:** † Helmholtz-Zentrum Berlin für Materialien und Energie GmbH, Hahn-Meitner Platz 1, 14109 Berlin, Germany; ‡ CNR-ISM, Consiglio Nazionale delle Ricerche, Istituto di Struttura della Materia, Via Salaria Km 29.3, I-00015 Monterotondo Stazione, Roma, Italy; § Department of Physics, University of Jaffna, Jaffna 40000, Sri Lanka; ∥ ENEA Research Center Portici, Piazzale Enrico Fermi 1, Portici 80055, Italy; ⊥ Dipartimento di chimica, dei materiali e della produzione industriale, Università degli studi di Napoli Federico II, Piazzale Vincenzo Tecchio 80, 80125 Napoli, Italy; # Department of Chemistry, Bielefeld University, Universitätsstrasse 25, 33615 Bielefeld, Germany

## Abstract

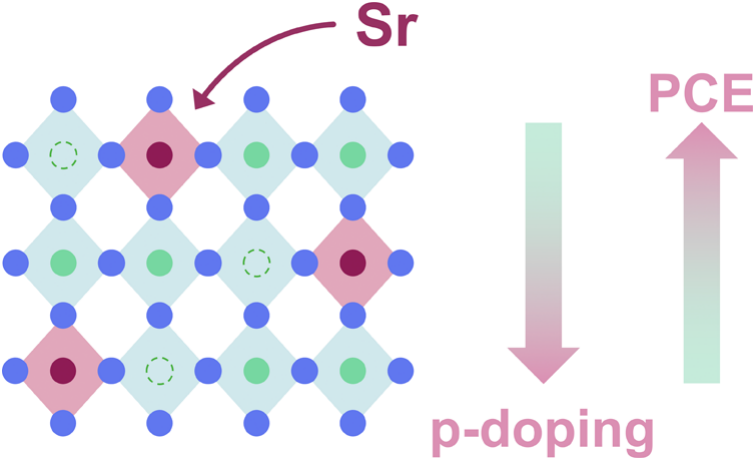

Tin-based perovskite solar cells offer a less toxic alternative to their lead-based counterparts. Despite their promising optoelectronic properties, their performances still lag behind, with the highest power conversion efficiencies reaching around 15%. This efficiency limitation arises primarily from electronic defects leading to self-p-doping and stereochemical activity of the Sn(II) ion, which distorts the atomic arrangement in the material. In this study, we investigate the effect of strontium doping in tin-based perovskite on the distortion of the material’s structure and its optoelectronic properties. Using a combination of Density Functional Theory calculations and experiments, we demonstrate that strontium doping reduces p-doping and structural strain. This approach improves the efficiency from 6.3% in undoped devices to 7.5% in doped devices without relying on dimethyl sulfoxide, a harmful solvent for tin-based perovskites. This method could enable precise control of tin off-centering and self-p-doping, advancing the development of efficient and stable tin perovskite solar cells.

Perovskite solar cells (PSCs) are one of the most promising technologies for realizing low-cost, easy-to-process, and efficient solar cells.^1,2^ The chemical flexibility of the crystal structure and, thereby, the tunability of the optoelectronic properties garner interest in studying them extensively, for both single junctions and tandem solar cells. So far, the record power conversion efficiency (PCE) is obtained with lead-based perovskite and has reached 26.1% (certified),^3^ approaching values obtained with silicon solar cells.^4^ However, since the toxicity and high bioavailability of lead (Pb) are well-known, the permitted amount of lead has constantly been reduced in the past few years, and for some applications, lead has even been completely banned from the market.^4−6^ Thus, lead toxicity is one of the main challenges hindering the successful commercialization of lead-based PSCs. To overcome this problem, less harmful, lead-free materials are being studied. Tin as Sn(II) represents the most promising alternative to lead due to its lower bioavailability.^5^ In addition, tin-based perovskite absorbers show advanced optoelectronic properties,^7^ such as high carrier mobility and high absorption coefficient, which are similar to or even better than the lead ones. However, PCEs reached with tin-based solar cells are still far behind those of their lead counterparts, and far from their theoretical efficiency of 32%, with a certified record PCE of 15.1%.^8^

The main limiting factors for tin-based perovskites are the high defect concentration due to Sn(II) instability and the *emphanisis* effect due to the presence of the lone pair on the Sn(II) cation.^9,10^ Contrary to Pb(II), Sn(II) tends to oxidize to the more chemically stable form, Sn(IV), leading to a self-p-doping of the perovskite film, which increases free charge recombination rate and harms the open-circuit voltage (*V*_oc_) and the overall efficiency of the device.^11^ This *emphanisis* is induced by the elements of the IV group (Pb, Sn, Ge), which show a tendency to have a stereochemical active *ns*^2^ electron pair, commonly called a “lone-pair expression”, generating an asymmetrical coordination environment where the metal is displaced from the center of the octahedron.^10,12,13^ This phenomenon is particularly prominent in lighter metals, where the higher energy of the *ns*^2^ level enhances the tendency of *sp* hybridization,^14^ which results in a hybridized orbital not symmetrical distributed around the atom like in Pb perovskite. The consequence is the formation of a tilted perovskite structure with asymmetrical octahedra,^13^ with no long-range order, hence not inducing ferroelectricity. This elucidates why the *emphanisis* effect poses a more significant challenge in tin perovskites than its lead counterpart.

Recently, much effort has been made to reduce tin oxidation, mainly using additives such as tin halides, SnX_2_ (X = F, Cl, Br) or hydrazine as potent reducing agents to compensate for tin vacancy.^15−17^ Moreover, in 2020, Pascual et al.^18^ and Saidaminov et al.^19^ reported independently that dimethyl sulfoxide (DMSO), the most common solvent used to prepare the tin-perovskite solution, is a source of oxidation itself, which necessitated research on alternative solvents and approaches controlling the crystallization of tin-perovskite^20,21^ While the reduction of tin oxidation has been an object of intense study, there is a lack of research on mitigating the active lone-pair expression of Sn(II). Recently, strontium iodide (SrI_2_) has been studied as a dopant in lead-based perovskites to reduce the defect concentration: the similar ionic radius allows the alkali metal to be incorporated into the crystal lattice without impacting the tolerance factor of the perovskite. Phung et al.^22^ have demonstrated the effect of Sr^2+^ on the electronic properties of lead perovskites, leading to a more n-type material when the alkali metal is incorporated into the lattice. Lately, the beneficial effect of Sr^2+^ doping has also been shown for tin and mixed tin–lead-based PSCs, improving the electronic quality of the film,^23,24^ However, no investigations have reported on the impact of the dopant on either the devices’ efficiency or their crystal structure. Notably, despite the crucial role of lattice stability in tin perovskites, tin off-centering remains a largely unexplored challenge with no systematic efforts made to effectively address or mitigate this distortion, leaving a critical gap in the development of stable, high-efficiency tin-based PSCs.

In this work, we aim to study the effect of SrI_2_ doping on tin perovskites and its role in preventing the *emphanisis* effect due to strontium’s similar ionic radius to tin and the absence of the lone pair. We studied the impact of Sr^2+^ on improving tin perovskite crystal structure and its microscopic and optoelectronic properties, significantly boosting solar cell device efficiency. Through a comprehensive analysis involving Density Functional Theory (DFT) calculations and practical experiments, we demonstrated that doping with SrI_2_ can be a successful approach to reduce both the self-p-doping and the off-centering of the Sn^2+^ in the octahedron, with a consequent enhancement of the PCE of the solar cells.

To examine the effect of Sr^2+^ doping in pure tin-based perovskite, we first fabricated devices with the p-i-n inverted architecture shown in Figure 1A. We tested their photovoltaic performance by adding SrI_2_ to the precursor solution. We investigated varying concentrations ranging from 0.1 to 2 mol % of SrI_2_ against Sn. Figure 1C–F reports the box plots of the photovoltaic parameters. The addition of SrI_2_ leads to an increase in PCE, with the champion device delivering a PCE of 7.5% obtained by doping the perovskite with 1 mol % of SrI_2_. The increase in the PCE is primarily attributed to the improvement in *V*_oc_ of an average of 70 mV and Fill Factor (FF) of 14%, the two photovoltaic parameters connected to the presence of defects inside the absorber material.

**Figure 1:**
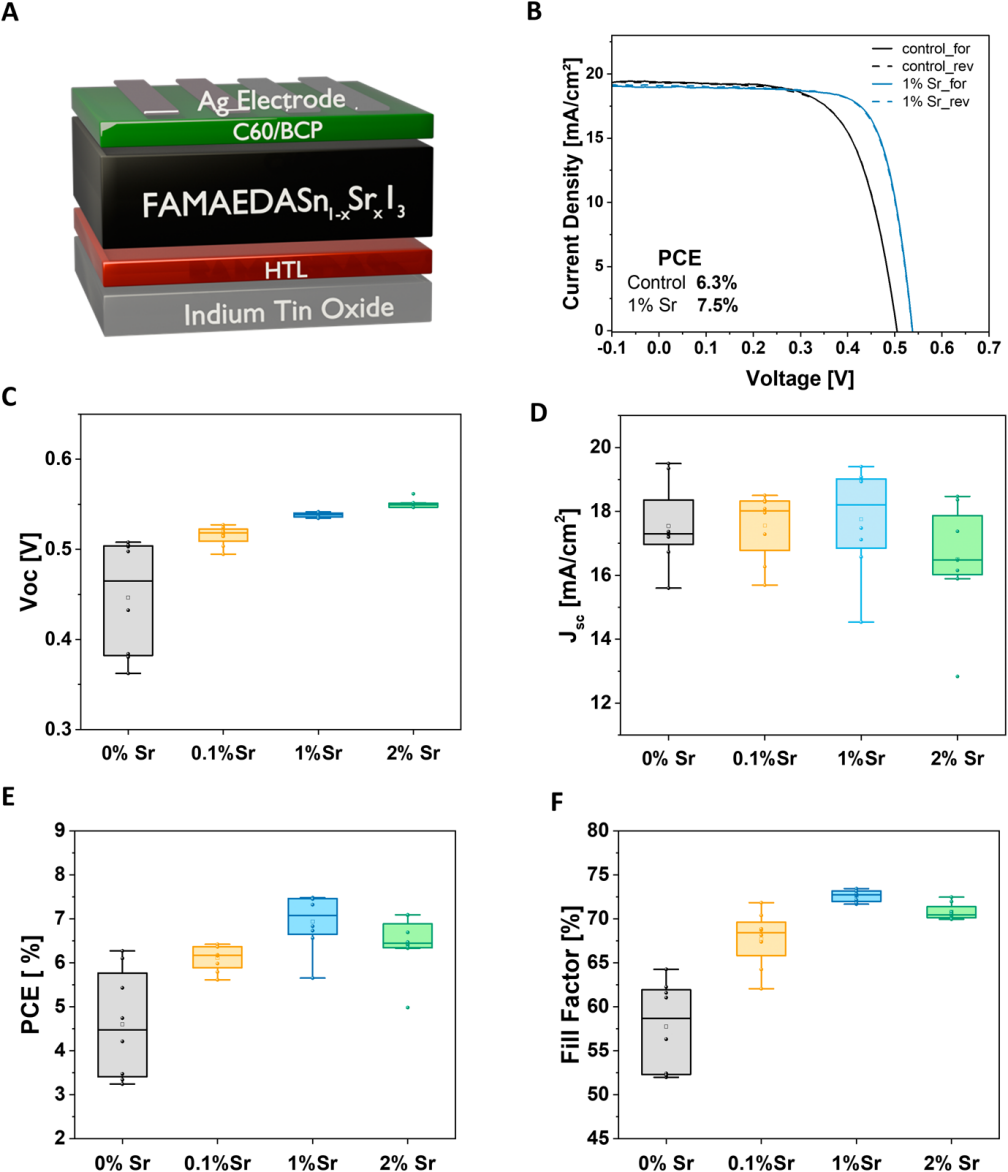
Photovoltaic performances of doped and undoped perovskite devices. A) Schematic illustration of the device architecture. B) *J*–*V* curves of the control sample and the best device, with 1% Sr. C–F) Box charts of the photovoltaic parameters at different doping concentrations.

After proving the beneficial effect of doping on the photovoltaic performances of the prepared devices, we further investigated the origin of the increase in *V*_oc_. To dive into the reasons behind the efficiency improvement through Sr^2+^ doping of the absorber film, we explored how the alkali metal dopant interacts with the crystal structure of tin perovskite by performing ab initio DFT calculations for Sr^2+^ incorporation into FASnI_3_. We considered Sr^2+^ substitution for both Sn(II) and FA^+^ cation sites (Sr_Sn_ and Sr^•^, in the Kröger–Vink notation) and Sr^2+^ interstitial positions in the perovskite crystal structure (Sr^••^) (Figure 2A–C)). Technical details of the calculation are given in the Supporting Information.

**Figure 2:**
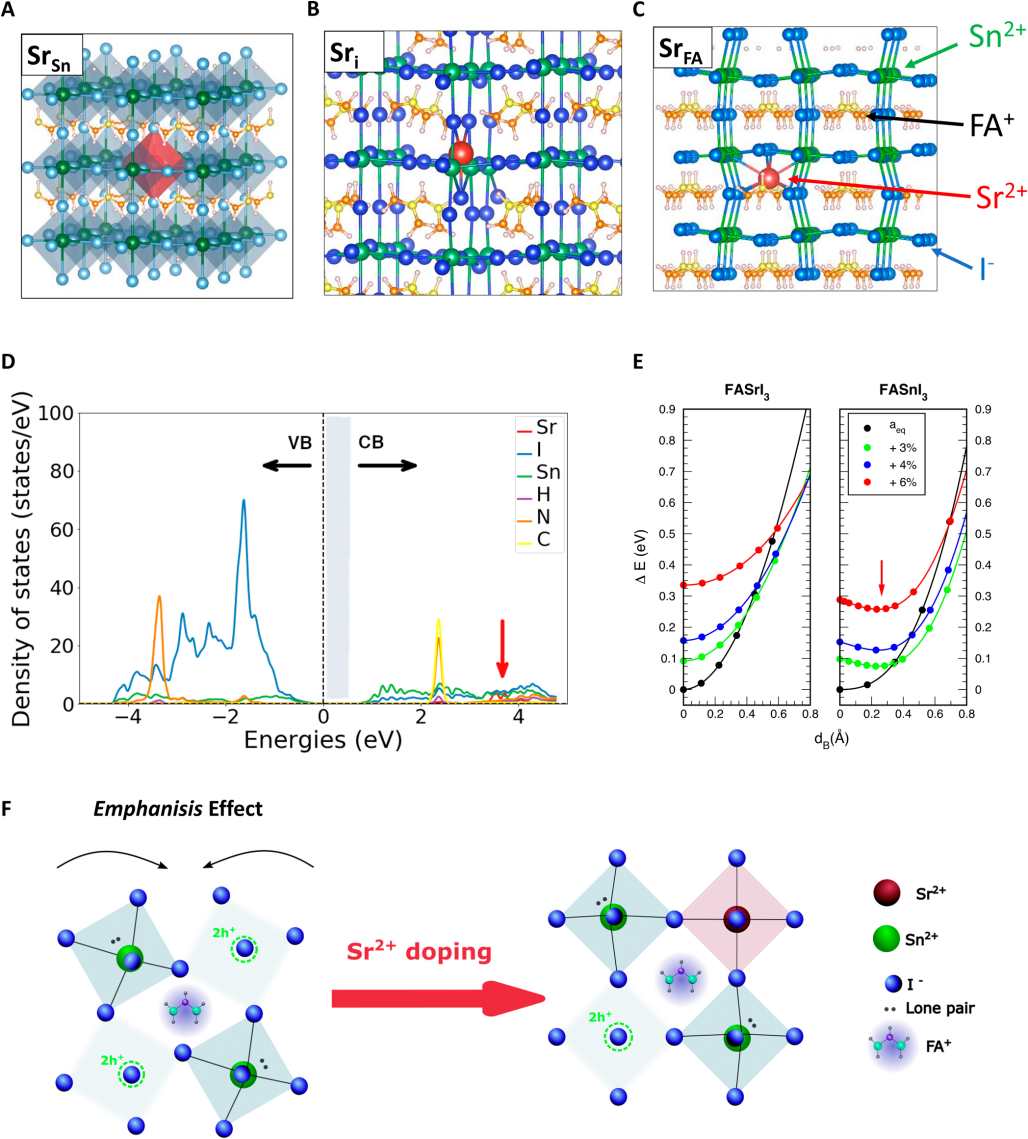
Interaction of Sr^2+^ in the crystal structure of FASI. A) Sn^2+^ substitution by Sr^2+^; B) Sr^2+^ in the interstitial position; C) Sr^2+^ substituting the A^+^ cation. D) DFT-GGA atom-projected density of states (pDOS) for substitutional Sr_Sn_. E) Energy landscape for cation off-centering displacement in FASrI_3_ and FASnI_3_ at various expanded lattice parameter *a*. F) Proposed healing mechanism of the tilting and tin off-centring upon Sr^2+^ doping.

To investigate possible mechanisms of incorporation of Sr^2+^, the energetics of both Sr-induced defects and intrinsic ones (Sn/FA/I vacancies and their interstitial counterparts) have been calculated at the generalized gradient approximation with the Perdew–Burke–Ernzerhof (GGA-PBE) level of theory. Formation energies *E*_f_[D*q*]{μ*_i_*, μ_F_} were computed for defects configurations D in different charge states *q*, assumed to be in equilibrium with atomic reservoirs at chemical potentials *μ_i_* and electron reservoir at the electronic chemical potential μ_F_.^25^ Variations of *μ_i_* values shift the equilibrium toward different possible chemical conditions (for instance, I-rich or Sn-rich ones). By limiting the chemical potential variations to equilibrium conditions in which FASnI_3_ is thermodynamically stable and no other segregated FA–Sn–I–Sr phases form, ranges of allowed values of *μ_i_* are restricted into a polyhedron in (μ_FA_, μ_I_, μ_Sn_, μ_Sr_) space (FigureS1A). Within this polyhedron, we chose two sets of chemical potential values representing I-rich (Sn-poor) and I-poor (Sn-rich) synthesis conditions and plot in Figure S1B,C the calculated formation energies of the stable charged states of the defects D, for possible *μ*_F_ values within the bandgap. DFT energetics indicate that it is energetically favorable for Sr^2+^ to sit on Sn(II) lattice sites, in this configuration acting as an isovalent substitutional defect: it is not active as a compensating dopant, as its empty electronic states are resonant in the conduction band (Figure 2D). A comparison of the electronic state of the undoped tin perovskite is reported in Figure S2. However, Sr_Sn_ may fill Sn(II) vacancies (*V*_Sn_^″^), dominant native defects in FASnI_3_ and sources of self-p-doping. DFT results also show that interstitials Sr_i_ and substitutional Sr_FA_ are both electron-donating defects, thus also possibly contributing to p-doping reduction (Figure S1B,C).

In the cubic phases of the perovskites, the *emphanisis* effect causes the metal cation to shift off-center within the octahedra. This phenomenon becomes more pronounced with increasing temperature,^10^ as the lattice expands. Ab initio calculations of energetics for both pure tin and pure strontium perovskite, as a function of different lattice parameters *a*, are shown in Figure 2E and Figure S3 for different lattice parameters varying up to 6% of the equilibrium value *a*_eq_. The lone pair effect is inherently connected to the electronic states of the metal cation and anion and only indirectly influenced by the monovalent cation.^10^ Nevertheless, we performed the DFT analysis for both Cs- and FA-based perovskite, finding in both cases that an energy minimum appears in Sn-based structures as the metal cation is displaced off-center. In contrast, no minimum is observed in CsSrI_3_ and FASrI_3_, suggesting that incorporating Sr into the crystal lattice mitigates the emphasis effect. The proposed healing mechanism is schematically represented in Figure 2F.

We then further analyze the effect of the doping on the morphological, optical, and electronic properties of the material. Scanning electron microscopy (SEM) first observed the thin film’s morphological quality. Figure 3A–D shows the SEM images of the film with doping level concentration varying from 0 to 10 mol %. When the alkali metal salt is added to the perovskite precursor solution, the grain size increases: 0.1 mol % Sr presents larger and more defined grains compared to the nondoped control samples, but the resulting film is inhomogeneous, exhibiting pinholes. When the concentration is further increased to 1 mol %, the grain growth is more enhanced, resulting in the coalescence of grains and, eventually, in the formation of a compact film ranging from an average diameter of 200 nm for the control to 300 nm for the target (1 mol % Sr), as shown in Figure S4. Nevertheless, a further increase of Sr^2+^ to 10 mol % leads to coagulation of the grains, worsening the film’s microstructure. The increase in the grain sizes reduces the grain boundary density, which, in turn, dramatically decreases the defect density and the nonradiative recombination.

**Figure 3:**
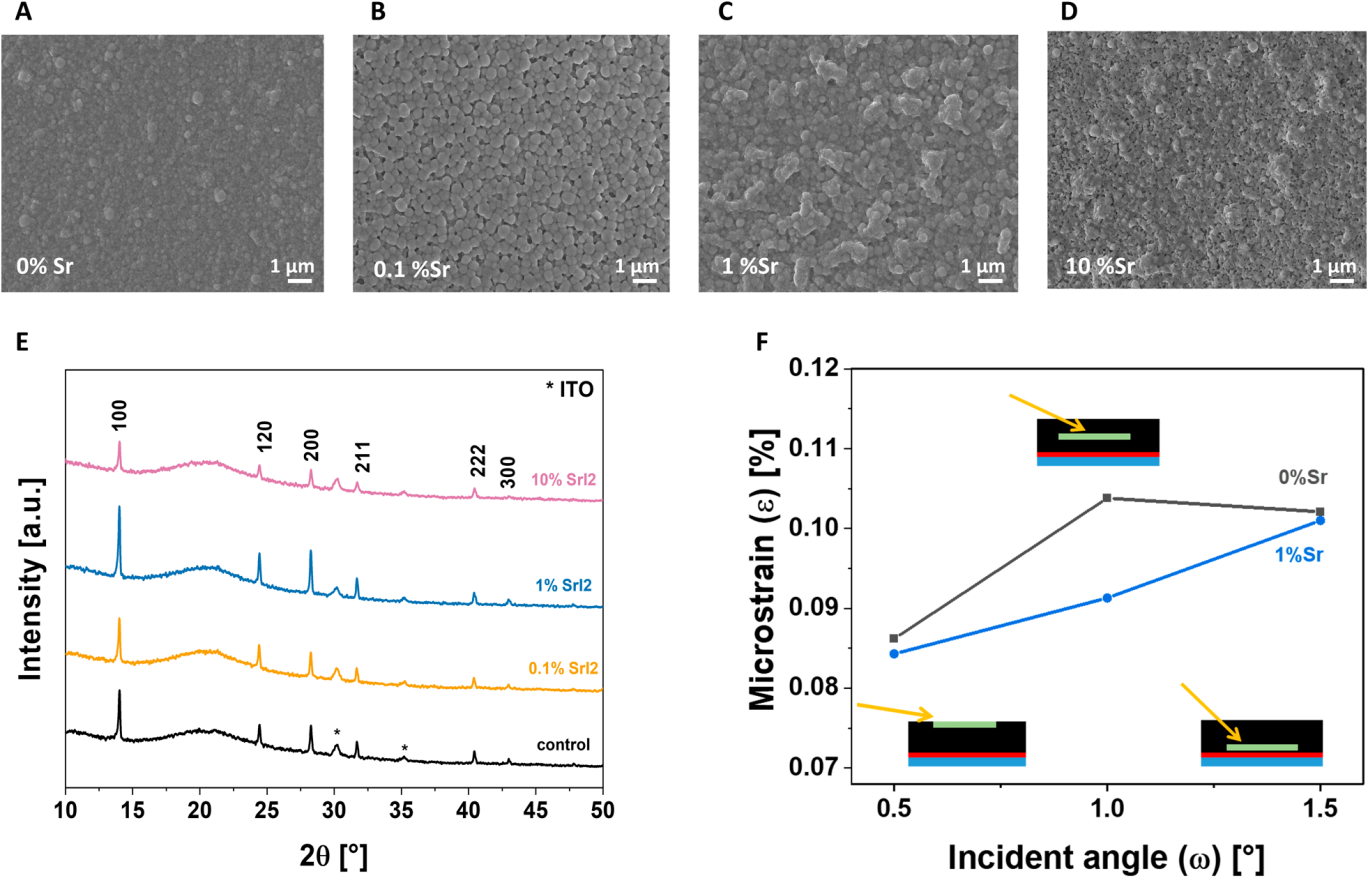
The effect of strontium doping on the morphology of the tin perovskite film. A-D) SEM images and E) Bragg–Brentano X-ray diffraction (BB-XRD) patterns of tin perovskite thin films with different concentrations of strontium. Peaks related to the ITO phase, marked with an asterisk, are evident in all the patterns. F) Depth profile of the microstrain values obtained via Le Bail refinement of grazing-incidence XRD (GI-XRD) pattern for the control and target concentration of Sr^2+^ and at different probing angles/depths.

The film crystallinity of samples with different amounts of SrI_2_ was investigated by using X-ray diffraction (XRD). The measurement was carried out under a protected atmosphere to avoid the degradation of the perovskite film. Diffractograms (Figure 3E) were recorded using Bragg–Brentano geometry, confirming the formation of tin-based perovskite. The position of the peaks remains unchanged with the addition of the dopant, a sign of the absence of macrostrain, which is consistent with the similar ionic size of the two metals, leaving the *d*_*hkl*_-spacing noticeably unchanged in the case of Sr^2+^ in the Sn(II) position. In addition, introducing Sr^2+^ does not generate new reflections, not even at the highest doping concentration of 10 mol % SrI_2_. Therefore, we conclude that no new phases are formed above the detection limit of XRD. In Figure S5, the Full Width at Half Maximum (FWHM) for the 100 peak is provided to measure the samples’ crystallinity. Including 0.1 mol % SrI_2_ shows no notable impact on the peak broadening. However, when the film is doped with 1 mol % SrI_2_, there is a reduction in FWHM, indicating enhanced crystallinity. Conversely, with 10% doping, crystallinity and peak intensity decrease significantly. While one might consider attributing the reduction of peak intensity to the introduction of a lighter atom (Sr) in the crystal structure, which possesses a lower scattering factor (*f*), combining SEM images and FWHM data suggests that the decrease in crystallinity is the more plausible explanation.

The combination of tilting of the octahedra due to tin vacancies and the off-center of the tin atom due to the *emphanisis* effect will distort the crystal structure, negatively impacting the final performance of the perovskite. The DFT calculation has shown that Sr^2+^ in the crystal structure can help fill the Sn(II) vacancies, helping to reduce the distortion in the lattice. To confirm this hypothesis, we use Le Bail refinement on grazing-incidence XRD (GI-XRD) to quantify microstrains within the lattice^26^ both with and without the introduction of the dopant. Microstrains are localized distortions in the crystal structure, leading to peak broadening.^27^ Since Le Bail refinement fits the entire pattern, we can quantify the broadening and, consequently, obtain values for the microstrain for each diffractogram. In GI-XRD, the X-ray penetration depth depends on the incidence angle ω.^28^ By varying ω during sample measurement, we gain insight into the microstrain evolution and the possible distribution of Sr^2+^ throughout the film depth. Control and target samples were both measured at ω equal to 0.5°, 1.0° and 1.5°, corresponding to probing, respectively, the surface, the bulk, and the interface with the hole selective contact (HSC) (Figure 3F). Based on the calculation, the microstrain values are decreasing after the addition of Sr^2+^ for all the incident angles analyzed, indicative of a decrease in defects and a more ordered structure.^29^ However, the decrease is slightly lower for the 0.5° and 1.5° angles. It is important to note that the microstrain value can be influenced by the incorporation of strontium and the presence of 1-D and 2-D defects. These defects are typically found with high densities in the topmost region and at the back interface (i.e., interaction between the perovskite film and the HSC), generating strain in the crystal structure that cannot be solely attributed to the effect of Sr^2+^ doping. Nevertheless, when analyzing the bulk of the perovskite, where these defects have a lower impact, the reduction in the microstrain is primarily related to the beneficial presence of Sr^2+^. These results align with DFT calculations, providing evidence that adding Sr^2+^ helps fill the vacancies and reduces the local distortions caused by lone pair expression.

To demonstrate the outcome of reducing *emphanisis* and defect concentration on the electronic and charge transport properties of the tin perovskite after the addition of strontium, we performed conductivity and photoluminescence (PL) measurements. The measure of conductivity in semiconductors can provide a direct and effective indication of doping and trap densities.^21,30−32^ Specifically, conductivity is proportional to the majority carrier density, which in tin perovskites is represented by holes, and the hole density is closely tied to trap density through the charge balance equation, where changes in carrier density reflect corresponding variations in traps. The results of the Hall conductivity shown in Figure 4A highlight the notable impact of the dopant on the electronic properties of tin perovskite, in which the conductivity is reduced by 2 orders of magnitude when 1 mol % of Sr is added to the perovskite solution. The reduction in conductivity can be therefore related to a decrease in the hole carrier concentration, which is in agreement with the DFT calculation, suggesting a decrease in Sn(II) vacancies by Sr^2+^ cations. We then performed PL measurements under transient and steady-state conditions to examine the effect of doping on the optoelectronic properties. Time-resolved photoluminescence (tr-PL) traces were fitted by using a biexponential decay equation. More details are reported in the Supporting Information. As shown in Figure 4B, for the target samples, the fast component of the biexponential decay (*τ*_1_), which is associated with nonradiative recombination,^32^ is slower compared to the control device Similarly, from the steady-state PL in Figure 4C,D, the target sample shows an increase in luminescence by a factor of 3 in the mapping photoluminescence compared to the control sample. The improvement in the photoluminescence properties, in both transient and steady-state conditions, shows the beneficial effect of strontium in reducing the defects in the bulk and surface of perovskite, one of the causes of nonradiative recombination. It is worth noticing that the PL peak position is also blue-shifted after adding the dopant, which could be due to an improvement in material crystallinity^33^ and a reduction of the tail defect state density.^34^ To rule out the possibility that this shift could instead arise from an increase in the bandgap due to strontium doping, we measured UV–vis spectra and calculated the optical bandgap using Tauc plots (Figure 4E,F). The fitted intercept of the linear approximation of the absorption edge with the *x*-axis yields an optical bandgap value of *E*_g_ = 1.39 eV for both the control and doped samples. This consistency indicates that the blue shift is primarily associated with the reduction of tail defects rather than changes in the optical bandgap. From the analysis of the PL and conductivity measurements, we therefore argue that the improvement of *V*_oc_ is mainly due to a decrease in nonradiative recombination due to a suppression of tin vacancies, also theoretically confirmed by DFT calculation.

**Figure 4:**
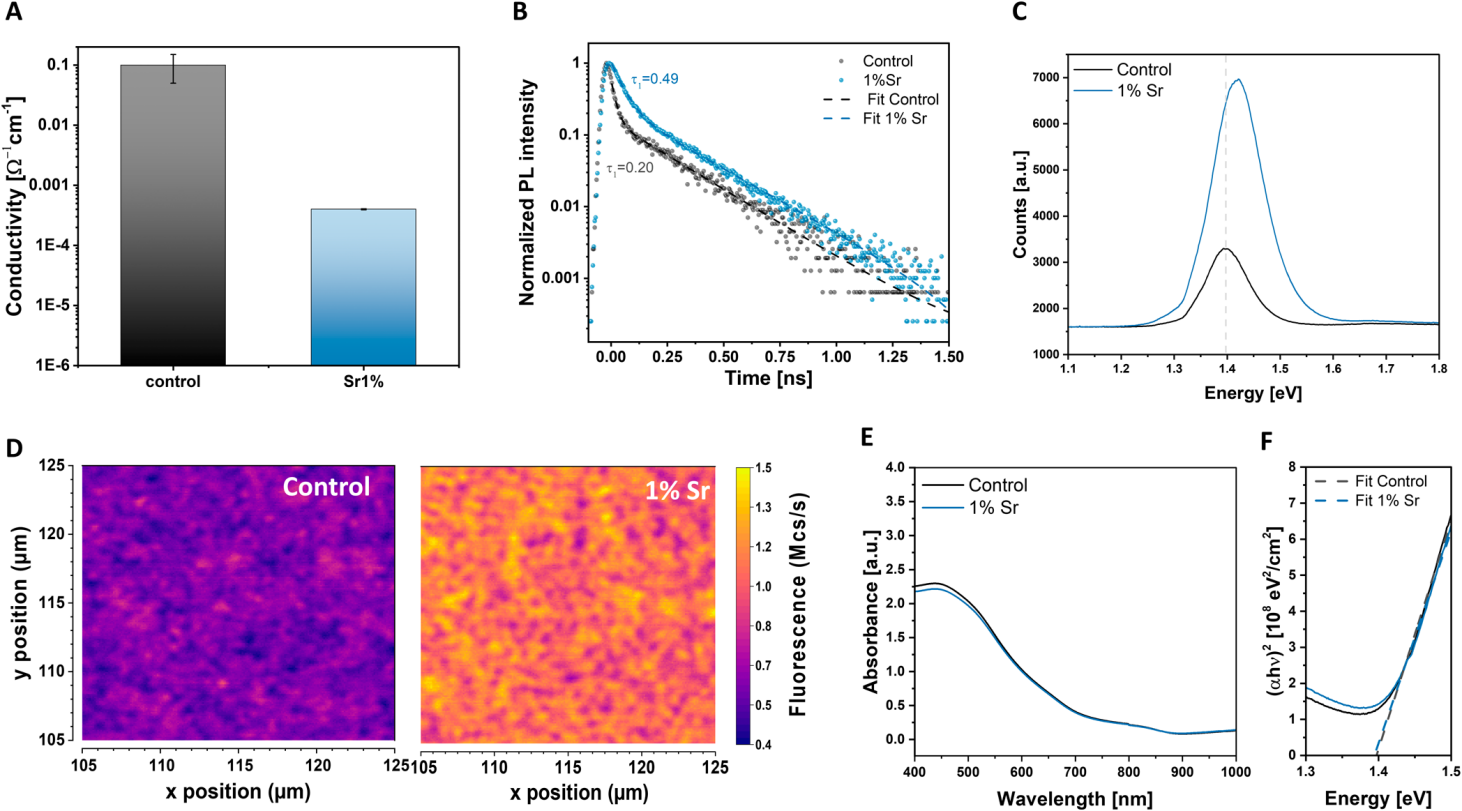
Enhancement of optoelectronic properties with strontium doping. A) Hall conductivity measurements. B) Tr-PL of target and control deposited on the glass. C) Steady-state PL and D) PL mapping of perovskite deposited on ITO. E) UV–vis absorption spectra and F) Tauc plot for band gap calculation of control and 1% strontium doping.

In conclusion, one of the main factors limiting the performance of tin-based perovskite solar cells is the instability of Sn(II), which creates vacancies, leading to a highly self-p-doped material and the presence of the *emphanisis* effect due to the lone pair on the Sn cation. Our research indicates that doping with SrI_2_ can address both of these issues. Sr^2+^ tends to fill the tin vacancies, reducing the hole concentration and decreasing the microstrain in the crystal structure. This reduction diminishes the amount of self-p-doping and the *emphanisis* effect, resulting in improved device performance, particularly in regard to *V*_oc_ and FF, resulting in an increase of PCE from 6.3% to 7.5%. This approach paves the way for controlling the tin off-centration and self-doping to achieve highly efficient tin-based PSCs. Furthermore, by incorporating Sr^2+^, we can enhance LEDs through a strategy based on the significant increase in PL intensity and charge transport properties.

## Data Availability

The data supporting the findings of this study are available within the article and in the Supporting Information.

## Supporting Information Available

Computational details, materials and methods, chemical potential diagram, pDOS, energy landscape, experimental details, histogram of grain sizes, FWHM of doped and control samples, example of Le Bail fitting of the XRD pattern, and TRPL decay curve fitting parameters (PDF)
