# Supplementary material for: Mitigation of Self-p-Doping and Off-Centering Effect in Tin Perovskite via Strontium Doping
Source: ACS Energy Lett. 2024 Dec 31;10(1):526–33. doi: 10.1021/acsenergylett.4c02974 (PMC11731519; doi:10.1021/acsenergylett.4c02974)
Supplement: Supplementary file 1 — nz4c02974_si_001.pdf [file nz4c02974_si_001.pdf]

Supporting Information

Mitigation of Self-p-Doping and Off-Centering Effect in Tin Perovskite *via* Strontium Doping

Chiara Frasca[1], Paola Alippi[2], Renè Schwiddessen[1], Karunanantharajah Prashanthan[1,3], Giuseppe Nasti[4], Shengnan Zuo[1], Muhammad Okash Ur Rehman[5], Mahmoud Hussein Aldamasy[1], Noor Titan Putri Hartono[1], Artem Musiienko[1*], Antonio Abate[1,5,6*]

[1]*Helmholtz-Zentrum Berlin für Materialien und Energie GmbH, Hahn-Meitner Platz 1, 14109 Berlin, Germany*

[2]*CNR-ISM, Consiglio Nazionale delle Ricerche, Istituto di Struttura della Materia, Via Salaria Km 29.3, I-00015, Monterotondo Stazione (RM), Italy*

[3]*Department of Physics, University of Jaffna, Jaffna 40000, Sri Lanka.*

[4]*ENEA Research Center Portici, Piazzale Enrico Fermi 1, Portici, 80055 Italy*

[5]*Dipartimento di chimica, dei materiali e della produzione industriale, Università degli studi di Napoli Federico II, Piazzale Vincenzo Tecchio 80, 80125, Napoli, Italy*

[6]*Department of Chemistry, Bielefeld University, Universitätsstrasse 25, 33615 Bielefeld, Germany*

Computational Details

Density Functional Theory (DFT) calculations have been performed within the Plane Augmented Wave (PAW) approach[1] with the Perdew-Burke-Ernzerhof (PBE) approximation[2] for the exchange correlation functional as implemented in the Vienna Ab-Initio Simulation Package (VASP).[3] The DFT-D3 method[4] has been used to include van der Waals dispersion correction terms. For $FASnI_3$, total energies of defects in different charge states q (D_q) have been calculated in supercells built up as (3x3x3) repetitions of the cubic unit cell and using a (2x2x2) k-point mesh. Structural optimization of defects geometries was performed until atomic forces are lower than 0.02 eV/Å. Defects enthalpies of formation have been calculated from total energies using standard formula[5] that involve the chemical potentials of the elements involved, $\mu_{Sr/Sn/I/FA}$, and the electronic chemical potential, μ_F. The finite-size corrections scheme of Freysoldt et al.[6] has been applied to correct for artificial interactions between periodic images of charged defects. The post-process analysis of defect formation energetics has been conducted with the *pydefect* code[7]. In particular, the allowed domain of chemical potentials μ_i values has been determined by imposing the chemical stability of the $FASnI_3$ phase (i.e., $\mu_{FA}+\mu_{Sn}+3\mu_I=H_f(FASnI_3)$) and excluding the formation of competing FA/Sn/I/Sr-containing phases. This translates in linear relations among the μ_i, of the form $k\mu_{FA}+j\mu_{Sn}+m\mu_{Sr}+n\mu_I < H_f(FA_kSn_jSr_mI_n)$, which are efficiently solved within *pydefect*. Formation enthalpy H_f of elemental phases of Sn, Sr, I, as well as of a few relevant compounds (SnI_2, SnI_4, SrI_2, FAI, SrSn, $SrSn_3$, Sr_3Sn_5), are calculated as total energies at the DFT-PBE level with consistent choice of PAW potentials and computational parameters chosen to assure convergence. For $FASnI_3$, this results in the region of admissible chemical potentials in (μ_{FA},μ_I,μ_{Sn}) spaced by the points A, B, C, D as in Figure S1A. Values of μ_i at the boundary points are also reported in the same Figure. In Figure S1 B-C defects formation enthalpies are plotted as a function of the electronic chemical potentials μ_F, (i.e., the Fermi level of the system, whose zero is set at the bulk valence band maximum) for chemical potential values representing I-rich (Sn-lean) and I-lean (Sn-rich) synthesis conditions. The general picture for cubic $FASnI_3$ is that intrinsic defects are mainly shallow: Sn- and FA-vacancies (V_{Sn}, V_{FA}) and interstitials iodine (I_{int}) are acceptors with transition levels below the VBM, while donor transition levels of the iodine vacancy (V_I) are located above the conduction band minimum. SnI_2 and FAI vacancies (V_{SnI2}, V_{FAI}) are always neutral. The lowest energy defect is the Sn vacancy, except in Sn-rich conditions where interstitial Sn is favored in *p*-type conditions. This picture is consistent with that for orthorhombic $FASnI_3$ phases[8]. Sr_{Sn} is a neutral defect, while stable charged states of Sr_{int} and

Sr_{FA} are positive, for any value of the electronic chemical potential. The calculated energetics shows that possible formation reactions for Sr_{Sn} (V_{SnI2}+SrI_2 -> Sr_{Sn}, Sr_{int}^{2+}+V_{Sn}^{2-} -> Sr_{Sn}) as well as for Sr^{+}_{FA} (Sr_{int}^{2+}+V_{FA}^{-} -> Sr_{FA}^{+}) are energetically favoured, as total energy differences are -1.16 eV, -0.145 eV and 1.14 eV, respectively.

We expect the prediction of shallow Sn- and I-related defects to be robust, as they get most of their character from the Sn-I bonding and antibonding band edges. The well-known DFT underestimate of fundamental gaps (0.5 vs. 1.4 eV experimental as for our results for cubic $FASnI_3$) may nevertheless lead to questions on the positioning of calculated levels in the gap. To clarify the issue, we used the Heyd-Scuseria-Ernzerhof (HSE) hybrid functional[9] for a sample defect case, the Sr_{Sn} substitution. Preliminarily, we determined the exact-exchange parameter needed to match the experimental gap to be α=0.5, a rather large value. The standard choice of α=0.25 leads only to some improvement (E_g=0.82 eV). We have checked Sr_{Sn} electronic structure with HSE calculations in a 2x2x2 supercell, finding an unchanged position of Sr-induced states (3.2 eV from the conduction band minimum) even with a bandgap value closer to the experimental one.

The pDOS of the undoped FASnI3 is reported in Figure S2. Please note that the corresponding atoms are depicted in different colours in Figure 2D and in FigureS2, for the doped and undoped pDOS, respectively. Compared to the doped pDOS, Strontium does not significantly alter the active region near the band gap in the tin perovskite structure and remains inactive within the gap region and up to ~2 eV above the conduction edge.

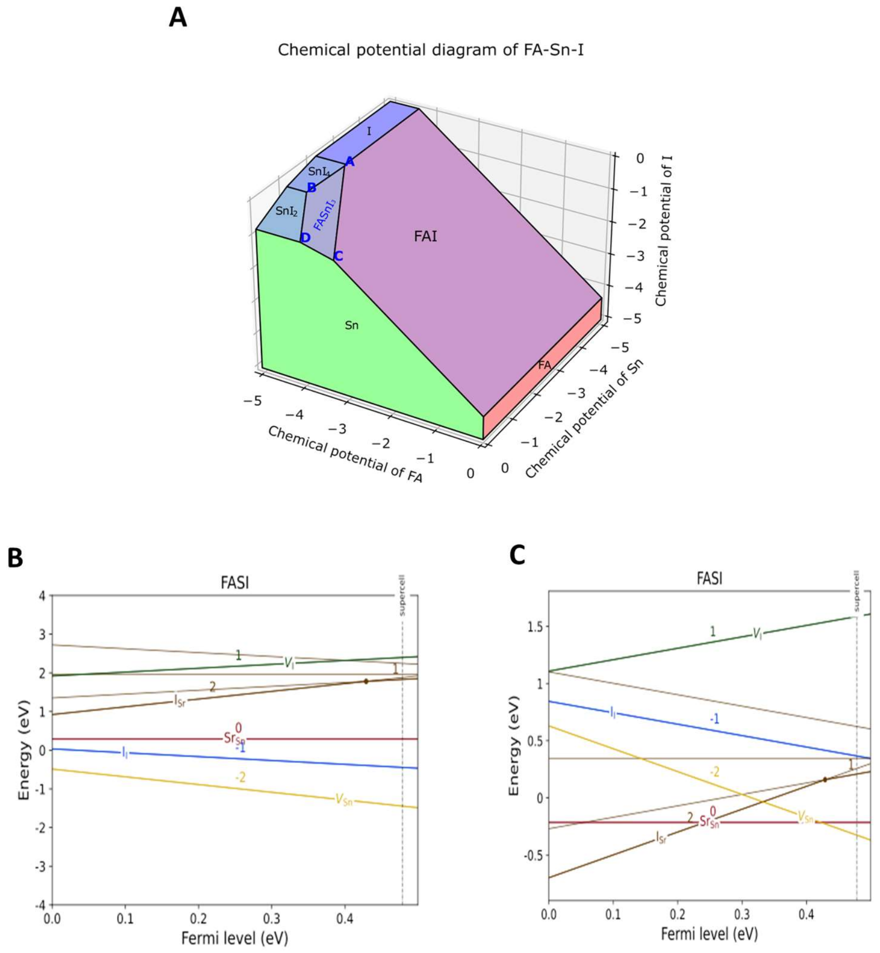

Figure S1, A) Chemical potential diagram of FA-Sn-I, with A B C D the vertexes of the polyhedron in which the perovskite phase is stable. Chemical potential values are in eV.; Formation energies for native and Sr^{2+}-related defects in cubic $FASnI_3$ as a function of the electron chemical potential, in **B)** Sn-lean and **C)** Sn-rich conditions. All energy values are in eV.

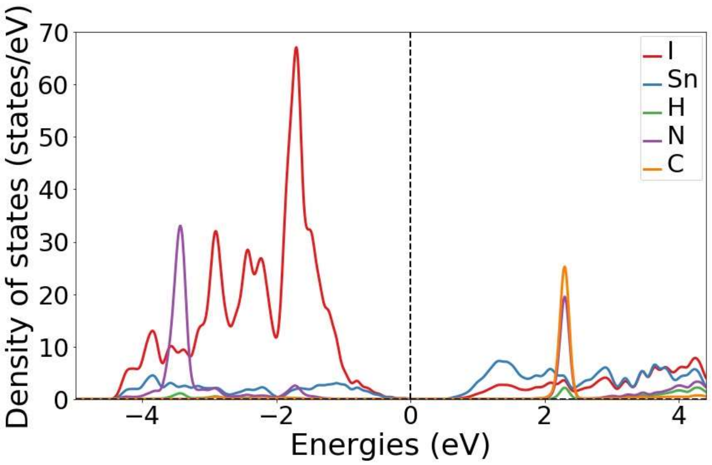

Figure S2 pDOS of bulk FASI, projected on Sn (blue lines), I (red lines), C, N and I (orange, violet, green lines, respectively) atoms.

For bulk cubic structures, an energy cut-off of E_{cut} = 400 eV is used and the Brillouin zone is sampled with a (12x12x12) Monkhorst-Pack grid; both values have been tested to give converged results. With this choice of parameters, the calculated equilibrium lattice constants of cubic phases are a_{eq}=6.32 Å ($FASnI_3$), 6.29 Å ($CsSnI_3$), 6.46 Å ($FASrI_3$), 6.51 Å ($CsSrI_3$). Total energies for B-cation displacements d_B from the center of the octahedra and along the <111> direction, shown in Figure 2e and S3 have been calculated at a=a_{eq} and at values expanded by 3%, 4% and 6%. At each expanded volumes, atomic forces are minimized to within 0.01eV/Å for d_B=0.

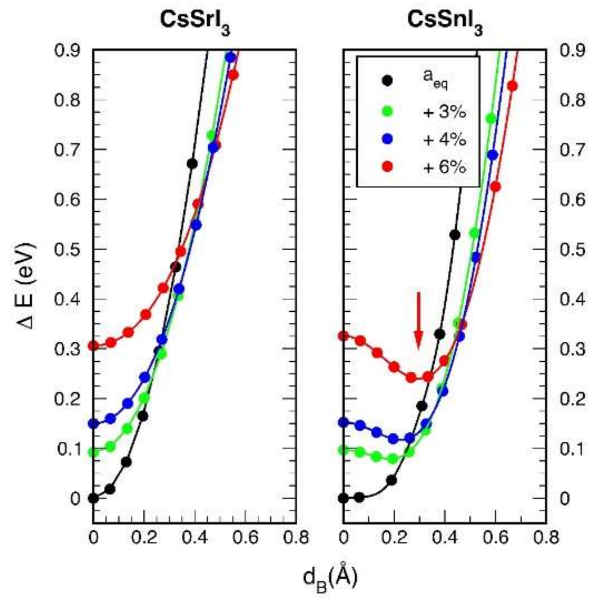

Figure S3 Energy landscape for cation off-centering displacement in $CsSrI_3$ and $CsSnI_3$ at various expanded lattice parameter *a*

Materials and methods

Perovskite solution preparation

The $FA_{0.78}MA_{0.2}EDA_{0.02}SnI_3$ perovskite solution with a concentration of 1 M was prepared dissolving 447 mg of SnI_2 (Sigma Aldrich, 99.99%), 165 mg of FAI, 33 mg of MAI, and 6.5 mg of $EDAI_2$ in 1 mL of DMF: DMI (6:1 v/v) solvent mixture. The solution was thoroughly mixed by shaking overnight at room temperature and then filtered using a 0.20 µm PTFE filter. Before spin coating, the solution was diluted with 4-(tert-butyl) pyridine (t-BP) at a 2:1 volume ratio. The doped samples were prepared similarly, by adding the proper volume of the Strontium iodide solution in the perovskite solution. SrI_2 is the sole additive used in the preparation of the perovskite solution. SnF_2 was excluded due to its limited solubility in the chosen solvent mixture.

Tin perovskite film preparation

100 µL of the as-prepared solution was spin-coated on ITO substrate covered with PEDOT: PSS thin film at a spin speed of 4000 rpm for 40 seconds, as HSC. 130 µL of p-xylene was used as an antisolvent and dripped onto the spinning substrate 15 s after the rotation began. The substrates were annealed at 100°C for 30 minutes. For the realization of the complete devices, 23 nm of C60 as Electron Selective Contact (ESC) and 8 nm of BCP used as hole blocking layer were sequentially evaporated on top of the perovskite film with a rate of 0.1 A/s, followed by 100 nm of Silver used as metal contact layer, with a rate of 1.0 A/s.

Device Characterization

The photovoltaic characterizations were conducted inside the glovebox using the all-in-one Arkeo measurement platform from Cicci Research Srl. The device's current density-voltage (J-V) characteristics were tested using a 12 LED sun simulator with a calibrated optical power density of 100 mW/cm^2. The measurements were performed with a scan rate of 100mV/s and a step size of 10 mV. The active area of the samples is 0.1 cm^2.

Scanning Electron Microscopy

The film morphology was investigated using a field emission Scanning electron microscopy (Zeiss Gemini 2.0) operated at 3kV.

In Figure S4, the grain size distribution of control and doped samples analysed with the ImageJ program is reported.

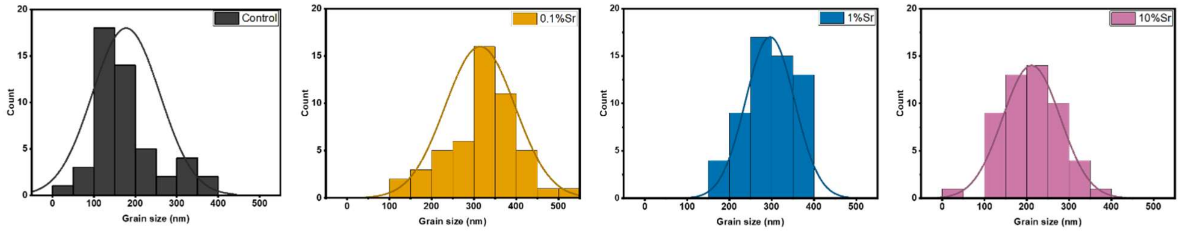

Figure S4 Histogram of grains sizes distribution of control and doped film

XRD Characterization

PANalytical X'Pert Pro MPD X-ray diffractometer with Ku-Ka radiation (l=1.54 Å) was used to collect the diffractogram for both Bragg Brentano and Grazing Incidence geometry. Samples were mounted inside the glovebox in a half-spherical dome, to keep them in an inert atmosphere during the measurements and prevent oxidation. An analysis of the crystallinity can be seen in FigureS5, the FWHM of each sample is reported.

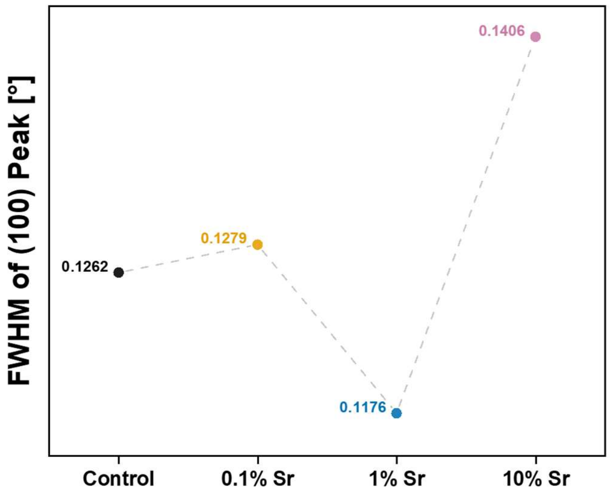

Figure S5. FWHM of doped and control samples.

Microstrain analysis

Le Bail refinement was utilized for the analysis of the microstructure. Contrary to Rietveld refinement, Le Bail can be used without prior knowledge of the atomic position in the crystal structure, which makes this technique more accessible. The analysis of the GI-XRD pattern was carried out using the software package FULLPROF Suite, which include the WINPLOTR program. The peak shape was modelled using the Thompson–Cox–Hastings (TCH) pseudo-Voigt function, which is a linear combination of the Lorentzian and Gaussian functions. The broadening of the peak is a combination of the material properties (microstrains and domain size) and instrumental broadening. To separate the two contributions, a standard reference material LaB6 was measured.

The pattern includes two phases, ITO and perovskite, which were treated separately in the refinement since they were not influencing each other. An example of the LeBail fitting is reported in Figure S6

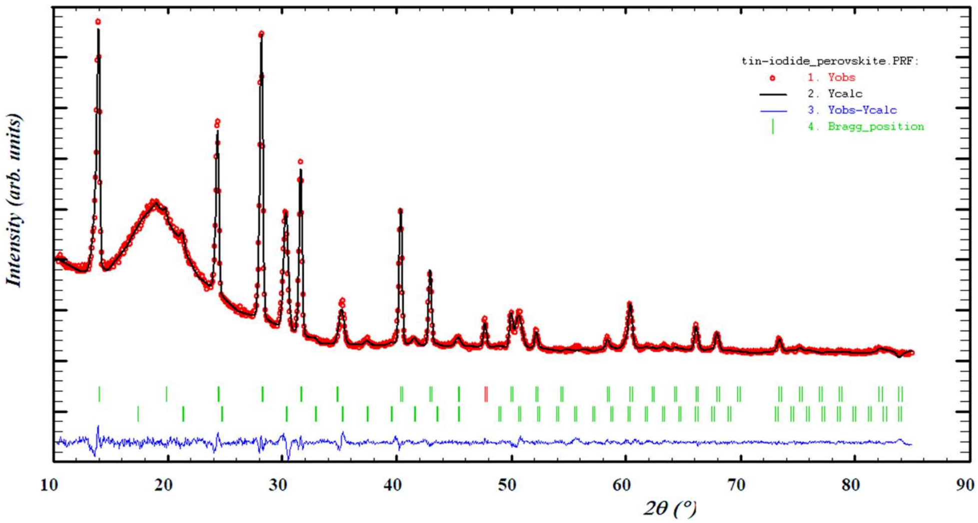

FigureS6. Example of Le Bail fitting of the XRD pattern. Red circles are measured data, black lines is the fitted pattern. The blu line in the bottom represent the residual between the observed data and the fitted value. The green stick are the Bragg position for the two phases, Tin perovskite and ITO.

Conductivity measurements

Conductivity of encapsulated tin perovskite thin films was probed using an AC Transport Controller Quantum Design model 7100 with a 4-probe measurement approach.

PL characterization

Steady-state photoluminescence (PL) measurements were performed in the air using a built-in house equipped with a 445°nm continuous wave laser (Insaneware) and an integrated sphere. The samples were previously encapsulated inside the glovebox applying an UV glue (*Bluefixx*) on the edge of the perovskite film and covering it with a cover glass (*Paul Marienfeld GmbH & Co. KG*), to protect them from degradation in the atmosphere.

The laser source was a NKT SuperK Fianium supercontinuum laser, with an adjustable repetition rate and user-selectable output wavelength band (Varia). Samples were measured using a home-built inverted confocal fluorescence microscope with a computer-controlled piezoscanner stage using the QuDI software suite. Excitation and detection were through the perovskite layer in confocal configuration using a low-magnification air objective and no imaging pinhole. Photoluminescence detection was via a silicon single-photon avalanche diode (Excelitas SPCM-AQRH-14) for PL mapping. Quasi-continuous wave measurements utilized a laser repetition rate of 78.2 MHz.

Time-resolved photoluminescence (TRPL) measurements were carried out on a home-built confocal PL setup utilising a 90:10 transmission:reflection beam splitter to separate the excitation and detection paths. Excitation was by a 700 nm diode laser (IB-705-B laser head with Taiko driver, Picoquant) with a pulse duration of 100 ps which could be operated in pulsed and cw modes. The laser beam was passed through a cleanup filter (FF01-700/13-25, Semrock) and the laser beam energy could be controlled by a computer-driven translation stage, which translated a linearly-graduated neutral density filter through the beam path. During PL transient measurements, the laser was operated at a pulse repetition rate of 100 kHz with a pulse energy density of about 40 $nJcm^{-2}$. Focusing and PL collection was by an off-axis parabolic mirror with 5cm focal length, the laser spot was a circle with a diameter of approximately 35 μm. Photoluminescence detection was by a silicon single-photon avalanche diode (Laser Components COUNT-50). The perovskite photoluminescence signal band was selected by a 715 nm longpass filter (FF01-715/LP-25, Semrock). For transient measurements, the PL count rate was collected by a TimeHarp260 Nano time-correlated single photon counting module (Picoquant).

The decay of the Transient PL was fitted using a bi-exponential function:

$y = A_1 exp(-x/\tau_1) + A_2 * exp(-x/ \tau_2) + y_0$

where $A_{1,2}$ and $\tau_{1,2}$ represent the amplitudes and lifetime of the component, respectively.

The values for the control and the target (1mol%Sr doping) are reported in Table S1

Table S1. Fitted parameters for TRPL decay curve

	**A_1**	**τ_1(ps)**	**A_2**	**τ_2 (ps)**	**τ(ps)**
**Control**	0.43793	20.07778	0.15487	228.348	74.48872631
**Target**	0.76929	48.91	0.2537	248.16	98.32370395

Supplementary References

(1) Kresse, G.; Joubert, D. From Ultrasoft Pseudopotentials to the Projector Augmented-Wave Method. *Phys. Rev. B* **1999**, *59* (3), 1758. https://doi.org/10.1103/PhysRevB.59.1758.

(2) Perdew, J. P.; Burke, K.; Ernzerhof, M. Generalized Gradient Approximation Made Simple. *Phys. Rev. Lett.* **1996**, *77*, 3865.

(3) Kresse, G.; Furthmüller, J. Efficient Iterative Schemes for *Ab Initio* Total-Energy Calculations Using a Plane-Wave Basis Set. *Phys. Rev. B* **1996**, *54* (16), 11169. https://doi.org/10.1103/PhysRevB.54.11169.

(4) Grimme, S.; Antony, J.; Ehrlich, S.; Krieg, H. A Consistent and Accurate Ab Initio Parametrization of Density Functional Dispersion Correction (DFT-D) for the 94 Elements H-Pu. *J. Chem. Phys.* **2010**, *132* (15). https://doi.org/10.1063/1.3382344.

(5) Freysoldt, C.; Grabowski, B.; Hickel, T.; Neugebauer, J.; Kresse, G.; Janotti, A.; Van De Walle, C. G. First-Principles Calculations for Point Defects in Solids. *Rev. Mod. Phys.* **2014**, *86* (1), 253–305. https://doi.org/10.1103/REVMODPHYS.86.253/FIGURES/24/MEDIUM.

(6) Freysoldt, C.; Neugebauer, J.; Van De Walle, C. G. Fully Ab Initio Finite-Size Corrections for Charged-Defect Supercell Calculations. *Phys. Rev. Lett.* **2009**, *102* (1), 016402. https://doi.org/10.1103/PHYSREVLETT.102.016402/FIGURES/3/MEDIUM.

(7) Kumagai, Y.; Tsunoda, N.; Takahashi, A.; Oba, F. Insights into Oxygen Vacancies from High-Throughput First-Principles Calculations. *Phys. Rev. Mater.* **2021**, *5* (12), 123803. https://doi.org/10.1103/PHYSREVMATERIALS.5.123803/FIGURES/5/MEDIUM.

(8) Shi, T.; Zhang, H.-S.; Meng, W.; Teng, Q.; Liu, M.; Yang, X.; Yan, Y.; Yip, H.-L.; Zhao, Y.-J. Effects of Organic Cations on the Defect Physics of Tin Halide Perovskites †. **2017**. https://doi.org/10.1039/c7ta02662e.

(9) Flores, M. A.; Orellana, W.; Menéndez-Proupin, E. Accuracy of the Heyd-Scuseria-Ernzerhof Hybrid Functional to Describe Many-Electron Interactions and Charge Localization in Semiconductors. *Phys. Rev. B* **2018**, *98* (15), 155131. https://doi.org/10.1103/PHYSREVB.98.155131/FIGURES/5/THUMBNAIL.
